# The facile conversion of waste biomass into few-layer graphene oxide

**DOI:** 10.1038/s41598-025-93037-x

**Published:** 2025-03-17

**Authors:** Rhoda Afriyie Mensah, Vigneshwaran Shanmugam, Elif Kaynak, Denis Sokol, Joel Wahl, Kim Cuong Le, Yang Zhang, Lin Jiang, Rasoul Esmaeely Neisiany, Emine Ayşe Turhan, Oisik Das

**Affiliations:** 1 Department of Civil, Environmental and Natural Resources Engineering, Luleå University of Technology, Luleå, 97187 Sweden; 2 Institute of Chemistry, Faculty of Chemistry and Geosciences, Vilnius University, Naugarduko 24, Vilnius, LT-03225 Lithuania; 3 Department of Engineering Sciences and Mathematics, Luleå University of Technology, Luleå, 97187 Sweden; 4 Division of Combustion Physics, Department of Physics, Lund University, P.O. Box 118, Lund, SE-22100 Sweden; 5 School of Mechanical Engineering, Nanjing University of Science and Technology, Nanjing, 210094 China; 6 Biotechnology Centre, Silesian University of Technology, Krzywoustego 8, Gliwice, 44-100 Poland; 7 Department of Polymer Engineering, Hakim Sabzevari University, Sabzevar, 9617976487 Iran; 8 Department of Material Science and Engineering, Koç University, Rumelifeneri Yolu, Sariyer, Istanbul, 34450 Turkey

**Keywords:** Wood waste, Catalytic graphitisation, Graphitic carbons, Few-layer GO, Engineering, Materials science

## Abstract

Carbon-based materials are highly sought after due to their superior properties, making them valuable for high-performance applications. However, most carbon-based materials are derived from fossil sources, and their synthesis often involves hazardous chemicals. Therefore, it is essential to develop sustainable methods for synthesising these materials from renewable resources, using fewer solvents, catalytic reagents, and generating minimal waste. In this study, few-layer graphene oxide (GO) was directly synthesised from waste biomass, without the formation of an amorphous intermediate, and its use as a fire retardant in two bioplastics was evaluated. Waste birch wood biomass was converted directly into graphitic carbon using manganese nitrate as a catalyst, with varying concentrations (0.003 to 0.1 mol-metal/g-wood) and treatment durations (1 and 2 h). The catalyst was doped through vacuum soaking and mild heating (90 °C), which facilitated the formation of graphitic carbon at relatively lower temperatures (< 1000 °C), eliminating the need for producing amorphous biochar prior to graphitisation. After pyrolysis at 900 °C and 950 °C for 2 h, the sample containing 0.005 mol-metal/g-wood, treated at 950 °C, exhibited the highest degree of graphitisation. This sample was further processed in a planetary ball mill with melamine as a dispersant for 30 min. Characterisation showed a broad absorption peak at 230 nm and the presence of semi-transparent sheets (3–8 layers), indicating the presence of GO. To evaluate its performance as a fire retardant, 2 wt% of the synthesised GO was added to polyamide 11 and wheat gluten bioplastics, which were then subjected to cone calorimeter tests. The results showed a 42% and 33% reduction in the peak heat release rate for polyamide 11 and wheat gluten, respectively, compared to their neat counterparts. The flame retardancy index further indicated that GO had a more significant impact on improving the fire safety of wheat gluten compared to polyamide 11. This study highlights a sustainable method for the preparation of few-layer GO at lower temperatures than contemporary methods, making the process more energy-efficient, environmentally friendly, and cost-effective. Additionally, the effectiveness of few-layer GO as a fire-retardant additive for enhancing the fire safety of bioplastics has been demonstrated.

## Introduction

In recent years, carbon-based materials have garnered significant attention due to their superior properties, including excellent chemical resistance, as well as thermal, mechanical, and electrical characteristics. These aforementioned properties have made them suitable candidates for various applications such as energy storage, nanocomposites, etc^1–4^. Amongst the different types of carbon structures, the most popular are graphene, carbon nanotubes, graphitic carbons, etc^5–9^. The ever-increasing need for these carbon-based materials has heightened the effect of their manufacturing on the environment^10^. The production of these carbon-based materials primarily relies on petroleum-based precursors, such as coke and coal. This constitutes one of the major drawbacks of the aforementioned materials because the base material is non-renewable and unsustainable in nature^11,12^. Therefore, it is critical to adopt a novel approach where the starting material is bio-based while the process is non-hazardous involving fewer solvents, more catalytic reagents, and generating minimal waste products. To decrease the dependence on petroleum products for developing carbon materials, biomass feedstock is being used as an alternative environment-friendly carbon source^13–16^. The advantages of biomass over petroleum-based counterparts are that it is renewable and economical as biomass can be converted to carbon at comparatively low temperatures. Additionally, the pyrolysis process for biomass conversion into carbon is considered to be carbon-neutral owing to the carbon emission balancing ability of the feedstock. Thus, high-value carbons (e.g. GO) can be engineered from renewable biomass, thereby propagating the concept of sustainable development.

GO is a material that holds great potential in various fields due to its unique properties. It is derived from graphene, a two-dimensional carbon allotrope by introducing oxygen-containing functional groups such as hydroxyl (-OH), ethers (-O-), and carboxyl (-COOH) groups onto its surface^17^. These functional groups impart various structural and chemical modifications in graphene, which results in the formation of GO. The presence of the functional groups gives GO its unique electronic and optical characteristics by creating defects and a bandgap in its structure, fostering the semiconductor nature of GO^18^. The oxygen groups on GO also provide sites for attaching other molecules, polymers, nanoparticles, etc. making it a versatile material for creating hybrid materials with tailored properties^19^. GO’s large surface area, flexibility, and strength make it useful for energy storage, sensing, and environmental applications^20^. Most 2D carbon materials, including graphene and GO, are typically derived from graphite as a precursor. Graphite is a naturally occurring carbon material, but its extraction and processing can have environmental implications^21^. As the demand for graphene-based materials continues to grow, there is a need for greener and more sustainable methods of graphite production.

Graphitic carbon has attracted considerable attention over the last decade due to its potential applications in supercapacitors, composite manufacturing, solar energy, etc^22,23^. Despite its diverse application potential, the main concerns associated with graphite production are the high-energy manufacturing methods (e.g. synthetic processes like the Acheson process, where silica is used at very high temperatures ca. 4150 °C)^24,25^. The conventional route to obtaining high-quality graphite is by exposure to elevated temperatures (~ 3000 °C) or stress graphitisation of feedstocks that are rich in carbon^26^. However, the disadvantages of these methods include very high costs, challenges associated with scaling up the process, the severity of the processing conditions, etc^5^. The utilisation of metal catalysts like Fe, Mn, etc. for the graphitisation process can lower the treatment temperature to below 1000 °C^27,28^.

Numerous biomass feedstock have been used by various researchers for producing graphitic carbons with specific graphitic order^29^. However, one of the major challenges in converting biomass to graphitic carbon is the amorphous nature of the feedstock, which requires a catalyst to induce the formation of graphitic structure at temperatures below 1000 °C^30–32^. Literature shows that iron, cobalt, and manganese are the best catalyst among different (like Ni, Mg, Ti, Cu, Cr, etc.) metals to produce graphitic carbon structures^5,33,34^. The mechanism behind this ordered structure formation in the presence of a metal catalyst is that the metal particle serves as a substrate for the carbon material to grow in an ordered manner^35^. Demir et al.^5^ studied the catalytic graphitisation of lignin using a two-step process consisting of both hydrothermal carbonisation and simple pyrolysis at 900–1100 °C. Firstly, lignin is hydrothermally carbonised at 300 °C and 103 bar pressure to generate biochar, which then undergoes subsequent pyrolysis in the presence of three different catalysts (nitrate salt of Fe, Mn, and Co) at 900–1100 °C in N_2_ atmosphere. A catalyst content of 0.083 mol-metal/g (for both Mn and Co catalysts) and temperatures of 900 and 1000 °C for Mn(NO_3_)_2_ and Co(NO_3_)_2_ respectively, were utilised. The results showed the obtained carbon was thermally stable and had good-quality graphitic carbon with both micro and mesoporous structures.

In another work, Major et al.^24^ analysed the catalytic graphitisation of miscanthus grass using a hybrid catalyst with both Iron (III) and Cobalt (II) nitrates. They stated that the highest degree of graphitisation was attained due to the formation of Fe-Co alloy nanoparticles in the system. A 1:1 ratio of cobalt nitrate moles to iron nitrate moles was taken to make the total moles of metal in the system 0.00718 moles. The authors also stated that the yield of graphitic carbon in the pyrolysed sample can be increased by raising the pyrolysis temperature. The results obtained allowed for the use of such renewable graphitised biochar in applications like catalysis, electronics, and composites with enhanced sustainability and economic benefits compared to conventional graphitic carbons. Although a lot of research has been done on catalytic graphitisation of biomass feedstock, the final product properties depend greatly on the extent of graphitisation of the carbon material.

The present study represents an important step towards developing a more sustainable and efficient method for producing two-dimensional carbon structures. Graphitic carbons were produced directly from biomass without an intermediate amorphous carbon step by impregnating the biomass with varying concentrations of manganese nitrate and pyrolysing at temperatures below 1000 °C. Low-energy intensive vacuum soaking and heating were adopted as efficient doping techniques to impregnate the catalyst into birch wood. The vacuum soaking and heating were not for the preparation of amorphous carbon, rather they were to dilate the pores of the wood for the catalyst to penetrate, thereby stimulating the efficient formation of graphitic carbon during pyrolysis and at lower catalyst contents. The aforementioned process also avoids the use of organic solvents for the doping of the precursor material. Manganese nitrate was chosen as the catalyst based on a prior study by Demir et al.^5^, which highlighted its superior ability to achieve graphitisation compared to other metal catalysts. Unlike iron and cobalt, manganese nitrate demonstrated greater efficiency in forming graphitic carbon at lower temperatures while minimising environmental impact, as corroborated by findings from Major et al.^24^. The resulting graphitic carbon was subsequently milled with melamine to produce few-layer GO.The current study advances existing literature by introducing a low-energy, scalable method that directly converts biomass into few-layer GO using manganese nitrate. This unique technique avoids intermediate biochar production, significantly lowering energy input and making the process more sustainable compared to conventional catalytic graphitisation methods. It also provides a means of producing bulk graphene, which can be used in a variety of applications, such as in the production of composites, batteries, and electronic devices. To explore the applications, the few-layer GO produced in this project was incorporated into wheat gluten and polyamide 11 plastics to investigate the effect on their fire performance using reaction-to-fire properties from cone calorimeter tests. This research promotes the sustainable production of carbon materials, aligning with green chemistry principles and advancing renewable resource utilisation.

## Materials and methods

### Materials

The biomass feedstock used for the catalytic graphitisation in this study was hardwood (birch), which is a widely available natural resource in Sweden that also naturally regenerates. Milled birch wood waste was sieved to a size of ca. 100–200 microns. The catalyst, Manganese (II) nitrate hydrate (98%) [Mn(NO_3_)_2_] (CAS No.: 15710-66-4), and melamine (CAS No.: 108-78-1) were purchased from Sigma Aldrich. Distilled water (DI) was used for all the wet procedures in the present work. The wheat gluten (WG) matrix for the composites was obtained from Lantmännen Reppe AB in Sweden and had a gluten protein content of 86.3 ± 0.3%, along with 0.9 ± 0.1% fat and 0.8 ± 0.1% ash. The powder form of Polyamide 11 (PA 11) was procured from Arkema in France.

### Doping of catalyst into wood

To increase the effectiveness of the catalyst in the graphitisation process, the catalyst, Mn(NO_3_)_2_, was first dissolved in DI water. For the doping process, 30 g of the wood particles were placed in Mn(NO_3_)_2_ solutions (1:10 g/g) prepared to achieve 0.003, 0.005, 0.083, and 0.1 mol-metal/g-wood. The wood particles were soaked in the Mn(NO_3_)_2_ solution under vacuum for 12 h. A pump was connected to the beaker containing the wood particles and the solution, and a magnetic stirrer was used to ensure thorough mixing. The pump evacuated the air from the beaker, creating a vacuum that facilitated the removal of air pockets from the wood’s pores, thereby enhancing the penetration of the catalyst. The vacuum soaking aided in pushing out pockets of air from the pores of the wood particles for easy penetration of the catalyst. The four catalyst concentrations were chosen for the study based on previous reports, where these are some of the lowest and highest amounts of catalyst used for graphitisation^5^.The wood particles for each concentration were filtered and doping was done by just heating in a beaker on a hot plate at temperatures between 80 and 90 °C for 1 and 2 h without the application of any pressure. The doped wood particles were kept in an air-circulating oven at 60 °C for 48 h to remove the moisture.

### Pyrolysis of catalyst-doped wood (Graphite production)

The moisture-free doped wood particles were initially pyrolysed at 900 °C in an inert atmosphere for 2 h in a macro thermogravimetric analyser (macro-TG reactor) using a nitrogen flow rate of 10 l/h. The catalyst-doped wood particles after pyrolysis were washed with dilute HCl, a few times, to remove the excess metal constituents (MgO salts) from the final product. The different samples prepared, along with their code names and processing conditions are shown in Table 1.

**Table 1:** Description of different pyrolysed samples and their coding.

Sample name	Amount of catalyst (mol-metal/g-wood)/description
1C	0.003/1 h heating time
1D	0.003/2 h heating time
2C	0.005/1 h heating time
2D	0.005/2 h heating time
3C	0.083/1 h heating time
3D	0.083/2 h heating time
4C	0.1/1 h heating time
4D	0.1/2 h heating time

After characterisation and analysis, the pyrolysis process was repeated for the samples with low catalyst content (1C, 1D, 2C, and 2D). For this step, the pyrolysis temperature was increased to 950 °C to increase the yield of graphite. The temperature, 900 °C, was optimised to 950 °C based on preliminary trials and aligned with thermal decomposition thresholds for catalytic graphitisation, as indicated by Major et al.^24^.

### Processing graphite to few-layer GO

The graphitic carbon from the selected sample 2D (0.005 mol-metal/g-wood heated for 2 h) was mixed with melamine at a ratio of 1:3, one part graphite, and 3 parts melamine according to the work of León et al.^36^. The graphite-melamine mixture was milled i.e., mechanically exfoliated in a planetary ball mill set at 100 rpm for 30 min. The milled graphite-melamine mixture was washed with distilled water heated at 60 °C to remove all traces of melamine, filtered, and dried at 100 °C for 30 min.

### Composite manufacturing

WG and PA11 were mixed directly with 2wt.% GO in a vortex mixer and 3 g of each mixture was moulded using Fortijne Presses TP 400 from the Netherlands. For samples based on wheat gluten (WG), the compression was performed at a temperature of 150 °C, a force of 290 kN, and a pressing time of 30 min. In the case of samples based on PA11, the compression was carried out at a temperature of 200 °C, a force of 250 kN, and a pressing time of 30 min.

### Characterisation of graphitic carbons

#### Scanning electron microscopy (SEM)

The samples were analysed in an FEI Magellan 400 field emission XHR-SEM instrument at an accelerated voltage of 3 kV and a current of 6.3 A.

#### X-ray diffractometry (XRD)

The XRD diffractograms of the powder samples were recorded by placing the sample in the holder and then recording the diffraction patterns using Cu kα radiation. The equipment (PANalytical EMPYREAN) is fitted with a PixCel3D detector and a graphite monochromator. The diffraction was measured by scanning 2θ from 0 to 120 °. The anti-divergence slit and anti-scatter slit were fixed at 1/8 and 1/4, respectively, during the analysis. The instrument was operated at 45 kV voltage and 40 mA current.

#### Raman spectroscopy

The relative amounts of graphitic carbon and disorder associated with the samples were analysed using Raman spectroscopy. Raman spectra of the powder samples were measured using a home-built Raman set-up, which was detailed in a previous work^37^. The samples were excited at a wavelength of 532 nm, with a power density of 6 × 10^3^ mW/mm^2^. Each spectrum is an average of four spectra with a total acquisition time of 400 s. Each sample was measured several times in both air and nitrogen environments to ensure there was no oxidation that could bias the results.

#### Nanoindentation studies

The hardness and reduced modulus of the samples were determined using a nanoindenter (Hysitron TI-950 tribo-indenter having a three-sided diamond Berkovich tip). The samples were mounted in epoxy set resin and then ground and polished after 12 h of curing. A standard quasi-static load function was used for a total of 12 indents on relatively flat sample surfaces found using an optical microscope with a 20 × objective magnification. Hardness and reduced modulus were calculated from the load–displacement data.

### Characterisation of GO

#### Ultraviolet–visible spectroscopy (UV–Vis)

The absorbance of the GO sample was measured with UV-Vis. A Hitachi UV-Vis U-1500 spectrophotometer was used for the experiment. The sample was dispersed in water and analysed in a 1 cm wide cuvette at a range of 200 nm to 400 nm.

#### Transmission Electron microscopy (TEM)

The morphology of the milled sample was observed in a 300 kV Hitachi HF-3300 S TEM equipped with a cold field emission emitter and energy-dispersive X-ray spectroscopy (EDX). The micrographs were taken at an accelerating voltage of 100 kV.

#### X-ray photoelectron spectroscopy (XPS)

The chemical states of the elements in the developed GO were examined via X-ray photoelectron spectroscopy (XPS) utilising a Thermo K-Alpha X-ray Photoelectron Spectrometer (Thermo Fisher Scientific, USA) under ultra-high vacuum. The binding energy scale was standardised with the C 1s peak, fixed at 284.8 eV. Data analysis and peak fitting were performed using the ‘Avantage software’ (version 6.6.0, Thermo Fisher Scientific).

### Cone calorimeter tests

The cone calorimeter tests were conducted using a TCC 918 cone calorimeter from Netzsch. The reaction-to-fire properties, peak heat release rate (PHRR), time to ignition (TTI), total heat release (THR), and time to peak heat release rate (TTPHRR) of the specimens were recorded from the tests and fire growth rate (FIGRA) and fire performance index (FPI) were calculated from the PHRR, TTI, and TTPHRR. The samples were exposed to a heat flux of 25 kW/m² until the flame was extinguished. The ISO 5660–1:2015 standard was employed for conducting the test.

## Results and discussion

### Characterisation of graphitic carbons

#### Scanning Electron microscopy (Morphology)

**Fig. 1:**
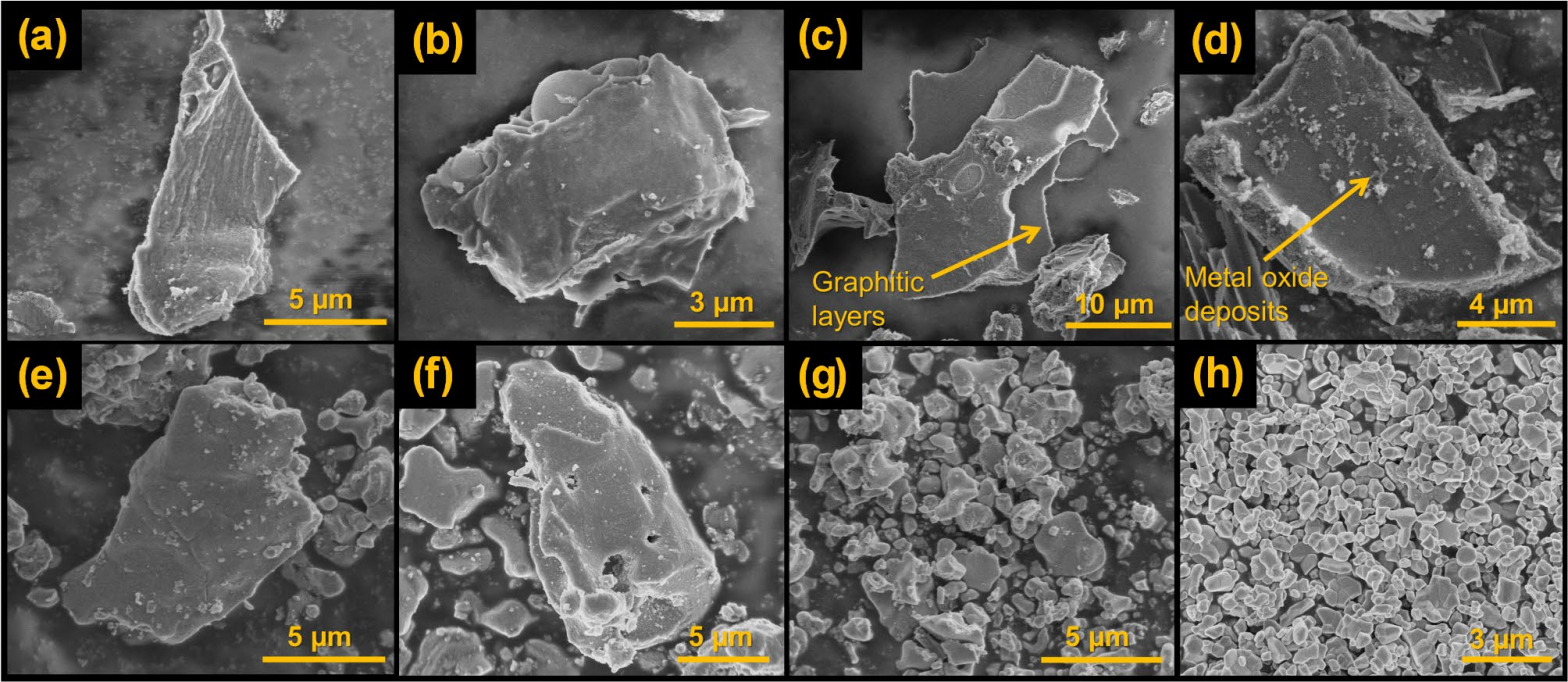
SEM images of pyrolysed samples (**a**) 1C, (**b**) 1D, (**c**) 2C, (**d**) 2D, (**e**) 3C, (**f**) 3D, (**g**) 4C, and (**h**) 4D.

Figure 1 portrays the representative SEM micrographs of the pyrolysed catalyst-doped wood. It can be observed that the doping of the catalyst has promoted the formation of three-dimensional graphite, especially visible in samples with a lower amount of catalyst (Fig. 1a, b, c, and d). The carbon structure in these samples has a flaked nature, which is also arranged in layers, analogous to a graphite structure. The doped samples with a higher amount of catalyst ( Fig. 1e, f, g, and h) also exhibit graphitic structure, however, these samples have a significant amount of metal oxide (MnO) coating the carbon surface. Hence, the samples containing a higher amount of catalyst display a metal-carbon mixed structure as also shown by Demir et al.^5^. From the SEM analysis, it can be stated that a low amount of catalyst (i.e., 0.003 and 0.005 mol-metal/g-wood) was apt for the generation of graphitic carbon without the presence of extra metal oxides remaining on the carbon surface. The high amount of catalyst (i.e., 0.083 and 0.1 mol metal/g), although can generate graphitic carbon, leaves behind some metal oxide deposits that consequently reduce the quality of the graphitic carbon.

#### X-ray diffractometry

**Fig. 2:**
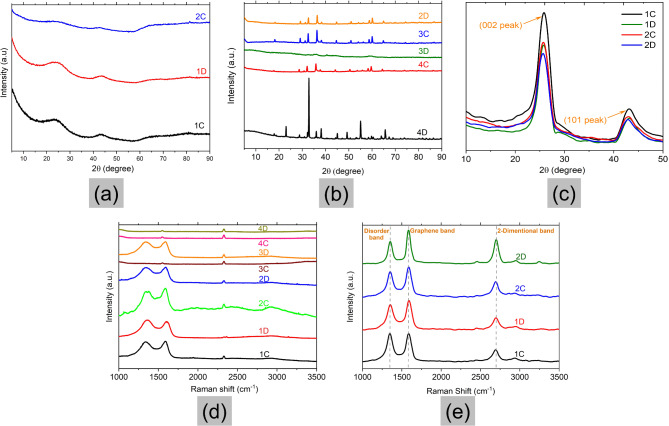
XRD and Raman spectrometry characterisation of carbon samples; (**a**) and (**b**) XRD results of catalyst-doped wood pyrolysed at 900 °C; (**c**) XRD results of catalyst-doped wood pyrolysed at 950 °C; Raman spectra of catalyst-doped wood pyrolysed at (**d**) 900 °C and (**e**) 950 °C.

Further confirmation and information on the graphitisation process and the extent of graphitisation can be achieved via XRD analysis. The XRD diffractograms of catalyst-doped wood pyrolysed at 900 °C are shown in Fig. 2a and b. It is observed that no characteristic peak of graphite (002 peaks at 2θ = 26^o^) can be seen in the XRD pattern. The samples with low catalyst concentrations (1C, 1D, and 2C) showed a very amorphous structure, which does not corroborate the SEM results (Fig. 1). The possible explanation for this observation is that the yield of graphite in the samples was very low, hence, only the amorphous particles were captured in the XRD. Only a broad hump can be seen in all the samples in Fig. 2a signifying the presence of large quantities of amorphous carbon compared to the crystalline equivalent (Fig. 2b). The reason could also be due to inefficient dispersion of the catalyst within the material leading to low-quality graphitic carbon. As already mentioned, catalytic graphitisation is predominantly efficient when a higher amount of amorphous carbon comes in contact with the metallic entity during the graphitisation process. Thus, during graphitisation, carbon comes in contact with the metallic part leading to good-quality graphitic carbon as the end product.

However, it should also be noted that with increasing catalyst content, the removal of the same becomes difficult and the peaks due to these metallic constituents can be noticeably seen in the XRD. The presence of these metallic impurities in the sample after washing interferes with the XRD analysis.

Interestingly, the samples with high catalyst content (2D, 3C, 3D, 4C, and 4D) shown in Fig. 2b displayed several characteristic peaks showing a crystalline structure. However, the peak with the highest intensity shifted from 26^o^ for graphitic carbons to 32^o^. These revealed that although the samples had a high crystallinity, the structures were different from those of graphitic carbons. This was also seen in the work of Demir et al.^5^ and could be attributed to the presence of MnO salts coating the surface of the samples.

For the (002) peak Scherrer equation (Eq. 1) was adopted to define the crystallite size of the graphite sheets in the crystalline samples, which are shown in Table 2.

1 $${L_c}=\frac{{k\lambda }}{{\beta \cos \theta }}$$

Where, *k* is a dimensionless shape factor with its value close to unity, *λ* is the wavelength of the X-Ray used, *β* is the line broadening at half the maximum intensity (FWHM) and *θ* is the Bragg angle. To determine the d-spacing values, Bragg’s law was used (Eq. 2).

2 $$2d\sin \theta =n\lambda$$

Where, *d* is the interplanar spacing, *θ* is the angle between the lattice planes and the wave vector of the incident wave, *λ* is the wavelength and *n* is the order of the reflection. The graphitisation degree parameter (*g*) was then calculated using the following equation:

3 $$g=\frac{{0.344 - {d_{002}}}}{{0.0086}}$$

Previous research has shown that graphitic carbons have a d-spacing ranging from 0.335 to 0.34 Å^34^. According to Table 2, the d-spacing calculated for all the crystalline samples was less than 0.335, which shows that the samples contain large quantities of salts.

To increase the yield of graphite, the experiments were repeated for the samples with low catalyst content (1C, 1D, 2C, and 2D), and the pyrolysis temperature was increased to 950 ^o^C. The X-ray diffractograms of the samples were obtained and the parameters were estimated using Eqs. 1–3. The results are presented in Fig. 2c; Table 3.

The curves in Fig. 2c show a (002) peak between 25.6^o^ – 26.3^o^ and a (101) peak at 43^o^ giving d spacing values ranging from 3.47 to 3.38 Å. According to Vlahov^38^, the d spacing of graphitic carbons is classified as fully ordered graphite (3.354 Å) to completely disordered graphite (3.440 Å). From Table 3, it can be inferred that samples 1C and 1D (with catalyst content of 0.003 mol-metal/g-wood) fall out of the range of graphitic carbons and have disordered graphite, however, 2C and 2D exhibit ordered graphitic structure with a graphitisation degree of ca. 58% and 70%, respectively.

**Table 2:** XRD data, d-spacing, graphitisation degree parameter, and crystallite size of catalyst-doped samples pyrolysed at 900 °C.

Sample name	2 Theta (degree)	d-spacing (Å)	Graphitisation degree	FWHM	Crystallite size (nm)
4D	28.8	0.309	0.398	0.20675	6.923887
4C	28.67	0.311	0.382	34.6696	0.041278
3D	29.11	0.307	0.436	–	–
3C	29.08	0.306	0.432	0.26766	5.351627
2D	29.19	0.305	0.445	0.26811	5.343978

**Table 3:** XRD data, d-spacing, graphitisation degree parameter, and crystallite size of low catalyst samples pyrolysed at 950 °C.

Sample name	2 Theta (degree)	d spacing (Å)	Graphitisation degree (%)	FWHM	Crystallite size (nm)
1C	25.66	3.47	–	0.92	100
1D	25.59	3.45	–	1.12	62
2C	26.20	3.39	58.12	0.75	96
2D	26.33	3.38	69.77	1.12	62

#### Raman spectroscopy

Generally, the Raman spectra of high graphitic materials such as graphite or multilayer graphene show three distinct peaks, namely the G band, D band, and 2D band due to in-plane vibrations. The G band, seen around ~ 1580 cm^[-1^, is associated with the in-plane bond-stretching vibrations of sp^2^-bonded carbon atoms. In a two-dimensional hexagonal lattice, it is a measure of the degree of graphitisation of the materials^39,40^. The D band at ~ 1350 cm^[-1^ is associated with the breathing mode of aromatic rings, which is activated by the presence of defects and edges in the materials. This peak area depends on concentration and types of defects^39^. The 2D band (~ 2700 cm^[-1^), on the other hand, is the D-peak overtone which does not require the defect presence. For few-layer graphene, this band is sensitive to the number of graphene layers present in the material^41^.

The results from the Raman experiments of samples pyrolysed at 900 °C are plotted in Fig. 2d. Most of the samples show D and G peaks in their spectra indicating the presence of the graphitic structure, while 3C and 4C show no graphitic structure. The widths of D and G are rather large (~ 100 cm^− 1^) and their second-order peaks (from 2400 cm^− 1^ to 3600 cm^− 1^) are very weak. The 2D peak cannot be observed. These features indicate disordered structures. There is a peak at ca. 2300 cm^− 1^, which is a result of the nitrogen in the air^42^. Sample 2C had the highest intensity counts, which is also supported by the distinct graphitic layers in the SEM images in Fig. 1.

The Raman spectra of the four selected samples with low catalyst concentration (1C, 1D, 2C, and 2D) pyrolysed at 950 °C are shown in Fig. 2e. The figure shows separated D and G bands with narrower bandwidths (a few tens of cm^[-1^) along with very distinct 2D peaks signifying the presence of graphitic carbons. Table 4 presents the peak positions and analysis made from the Raman spectra.

**Table 4:** Positions of D, G, and 2D bands and analysis from the Raman spectra.

Sample	Position of D band (cm^−1^)	Position of G band (cm^−1^)	Position of 2D band (cm^−1^)	I_D_/I_G_	Number of graphitic layers
1C	1353	1585	2691	1.0	1.7
1D	1350	1584	2691	0.84	2.8
2C	1354	1583	2695	0.82	3.9
2D	1354	1584	2701	0.62	3.0

The number of graphitic layers was calculated using Eq. (4).

4 $${w_G}=1581.6+\frac{{11}}{{1+{n^{1.6}}}}$$

Where *w*_*G*_ is the G band position in wavenumbers, and *n* is the number of layers present in the sample. The I_D_/I_G_ ratio obtained from the Raman spectra can be used as an effective tool to distinguish the extent of graphitisation and defects in a material, in particular, the size of the basic graphitic structural unit is inversely proportional to I_D_/I_G_^43^. The lower the I_D_/I_G_ ratio, the lesser the defects or disorders associated with the system, and depicts a higher amount of crystalline or graphitic content in the final product^44^.

It can be seen in Table 4 that the intensity ratio, I_D_/I_G_, decreases with increasing catalyst concentration and heating time. Sample 2D had the lowest intensity ratio depicting lower defects in the graphitic structure, corroborated by the highest intensity 2D peak in Fig. 2e. The estimation of the number of graphitic layers showed that all the samples displayed more than one layer of graphene.

From the Raman and XRD results, sample 2D (0.005 mol-metal/g-wood) displayed the best graphitisation degree and lowest defects in the graphitic structure, hence, this sample was chosen for further analysis.

### Nanoindentation

**Fig. 3:**
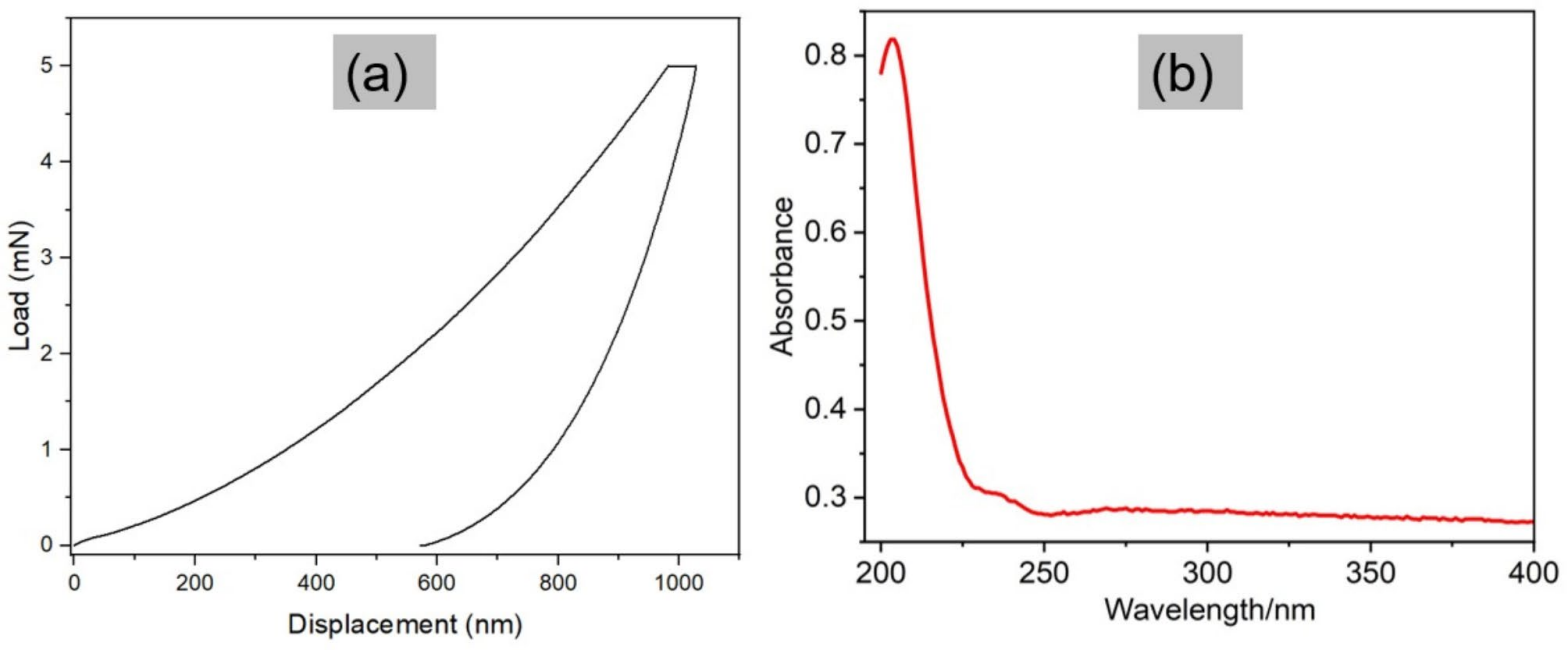
(**a**) Nanoindentation results (load versus displacement curves for 2D graphite sample). (**b**) UV–Vis results for sample 2D.

Nanoindentation tests were performed on the 2D graphite sample to measure its mechanical properties, hardness, and modulus. A Berkovich indenter applied a controlled force to the graphite, creating a small indentation on its surface. By measuring the force and displacement of the indenter, the mechanical properties were calculated^45^. Graphite is a layered material consisting of stacked sheets of hexagonally arranged carbon atoms. Within each sheet, the carbon atoms are covalently bonded, however, there are weak Van der Waals forces between adjacent sheets, which allow them to slide past one another. This weak interlayer bonding makes graphite relatively soft and deformable in the direction perpendicular to the sheets, while being very stiff and strong in the plane of the sheets^46^. The results obtained from the nanoindentation tests are as follows (Fig. 3a); Hardness: 0.24 ± 0.02 GPa and reduced modulus: 4.84 ± 0.26 GPa. On the other hand, biochar, an amorphous carbon, made at 900 ℃ (where pinewood feedstock was used), has been reported to have higher hardness of ca. 4 GPa^14^. Thus, the hardness of the graphite from sample 2D is lower than that of biochar, albeit from different biomass (pine). This signifies that, in general, biochar has a high resistance to indentation and plastic deformation compared to graphite. The catalyst impregnation and higher treatment temperature aided an increase in the degree of graphitisation of the biomass. The layers of the graphitic sheets allowed the slipping of the basal planes during indentation, leading to a reduction in the hardness values. The lower hardness value of graphite compared to biochar also reflect the increased graphitisation degree, which aligns with the findings of McDonald-Wharry et al.^44^, suggesting structural evolution toward layered graphite weakens interlayer bonding, unlike biochar’s dense amorphous structure.

### Production of GO

Few-layer GO was produced by adopting the mechanical exfoliation method. In this method, the graphite with the highest graphitisation degree (2D) together with melamine was milled in a planetary ball mill to separate the layers using mechanical force. The melamine acted as a dispersant that aids the graphene flakes from re-aggregating. Additionally, the melamine reduced the surface tension between the graphite flakes making it easier to separate.

During the milling process, the melamine molecules are inserted between the layers of graphite in a process called intercalation. This leads to the expansion of the graphite layers resulting in easier separation into individual graphene layers. The as-produced few-layer GO was analysed using Ultraviolet-Visible Spectroscopy, Energy Dispersive X**-**Ray Spectroscopy, X-Ray Photoelectron Spectroscopy, and Transmission Electron Microscopy methods.

#### Ultraviolet–Visible spectroscopy

The UV-Vis technique measures the absorbance of light at different wavelengths by a sample, which can provide information on its electronic and optical properties. Graphene dispersions typically show an absorbance peak around 210–300 nm due to π-π * transitions of the sp^2^ domains^47^. The result from the UV-Vis test of the milled 2D sample is shown in Fig. 3b. The fluorescence pattern found between the wavelengths of 200 to 250 nm is similar to the results for GO dispersions found in other studies^48^. Figure 3b shows a peak at ca. 230 nm, which is a result of aromatic C-C bonds in GO^49^. It was, however, noticed that the absorption peak is not very sharp but rather spread out. This broadening shows the presence of different electronic transitions happening within the material due to GO having a somewhat disordered structure and the different oxygen functional groups attached to it. These factors create variations in the energy levels of the electronic states, resulting in a broader range of absorption wavelengths. Additionally, the introduction of oxygen functional groups and defects disrupts the π-conjugation within the graphene lattice, resulting in a decrease in the bandgap and a shift towards longer wavelengths^47,50^. Based on the comparison from literature, it can be inferred that the graphite was successfully converted to GO. Further investigations were carried out with Energy-dispersive X-ray spectroscopy (EDX), X-ray photoelectron spectroscopy, and TEM.

#### Energy dispersive X-Ray spectroscopy (EDX)

**Fig. 4:**
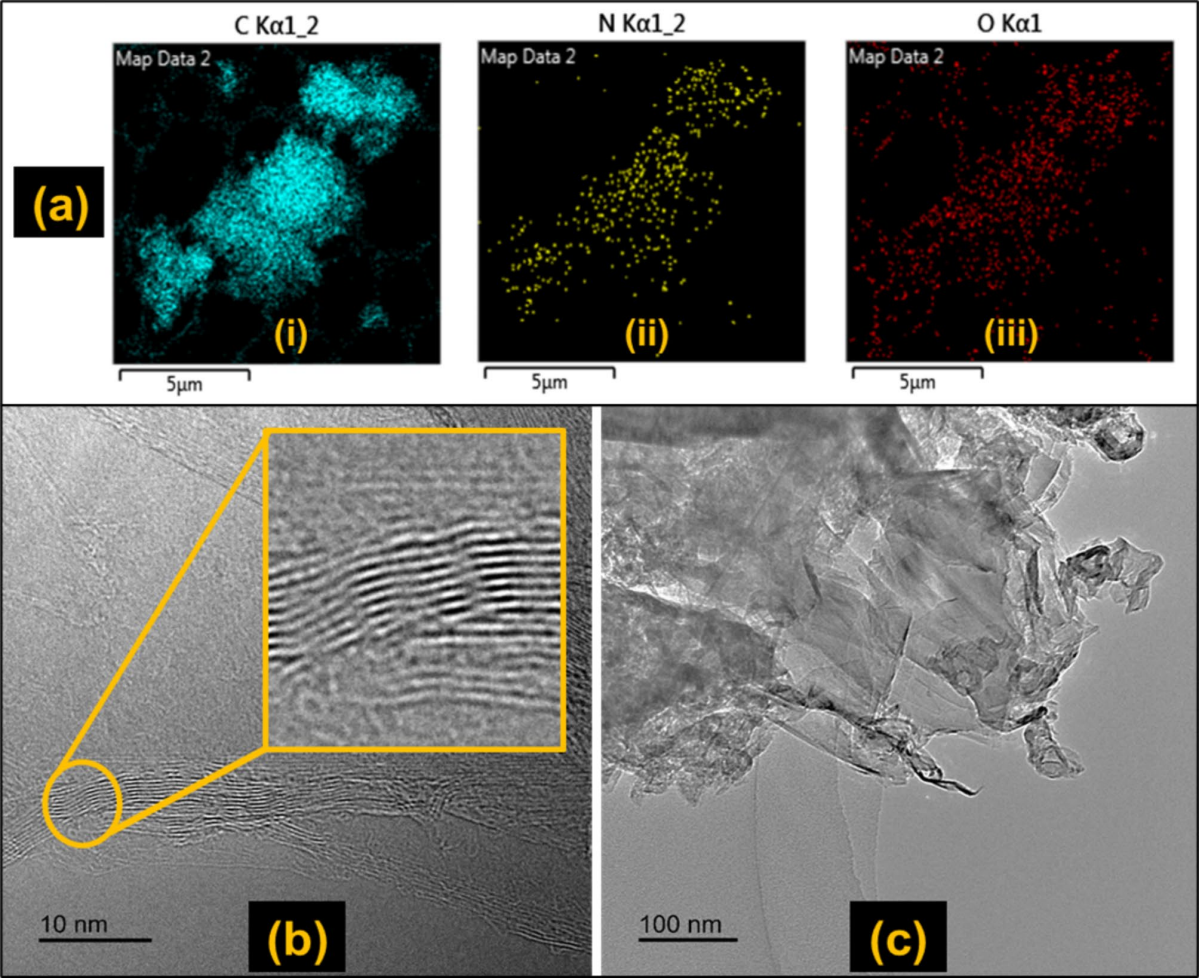
(**a**) EDX elemental map of milled 2D sample; TEM micrographs of the milled 2D sample showing (**b**) the stacked layers, wrinkles, and folds (**c**) the semi-transparent nature similar to that of GO.

EDX analyses the elemental composition of samples by providing information on the types of elements present in a sample and their relative abundance. This technique was used to characterise the milled 2D sample to determine its composition. Figure 4a shows the EDX map of the sample whereas the elemental constituents with their corresponding weight percentages are listed in Table 5. The results show that the sample contains ca. 91% carbon, 5% nitrogen, and 4% oxygen. The EDX results clearly reveal the presence of oxygen in the sample, indicating that the graphene was oxidised during milling/processing as confirmed in the work of Mahmoud et al.^51^, where the ball milling time for processing graphite into graphene affected the degree of oxidation of the end product. The nitrogen element identified could be attributed to the presence of nitrogen-containing functional groups or impurities during the synthesis or oxidation process. It should be noted that the presence of nitrogen in GO can enhance its properties and introduce additional functionality such as increasing the charge carrier density and facilitating better electrical conductivity. Nitrogen atoms can also serve as active sites for redox reactions, improving the charge storage capacity and overall performance of supercapacitors and lithium-ion batteries^52^.

**Table 5:** Elemental composition of sample 2D from EDX.

Element	Wt%	Absorption correction
C	90.45 ± 1.8	1.2
N	5.20 ± 1.3	4.6
O	4.34 ± 0.55	2.7

#### Transmission Electron Microscopy (TEM)

The morphology and internal structure of the milled 2D sample were observed using TEM. In the TEM, GO sheets have distinctive characteristics. They show irregular shapes (edges) and sizes with variable thicknesses due to the oxidation process^53^. The micrographs of the milled 2D samples are illustrated in Fig. 4b and c. Figure 4b shows the irregular and stacked layers of the GO, which was achieved as a result of the impact forces generated from the collisions of the balls in the planetary ball mill that allowed for the sliding of the graphite particles. Some wrinkles and folds as well as the variation in layers (from 3 to 8 layers) are present in the structure, which are caused by the introduction of oxygen functional groups. The number of layers of the GO from the current study is similar to a commercial GO from GrapheneXpert, Iran that had 8 layers as determined through reflection electron energy loss spectroscopy by Stobinski et al.^54^. An estimation of the spacing between the layers in Fig. 4b revealed a distance of ca. 0.34 nm. Some regions of the sample were more compact due to aggregation of the particles. Figure 4c shows a semi-transparent structure akin to GO micrographs reported by Aziz et al.^55^.

Following the results from UV-Vis, EDX, and TEM, it is evident that few-layer GO has been successfully produced from waste birch wood.

#### X-ray photoelectron spectroscopy (XPS)

**Fig. 5:**
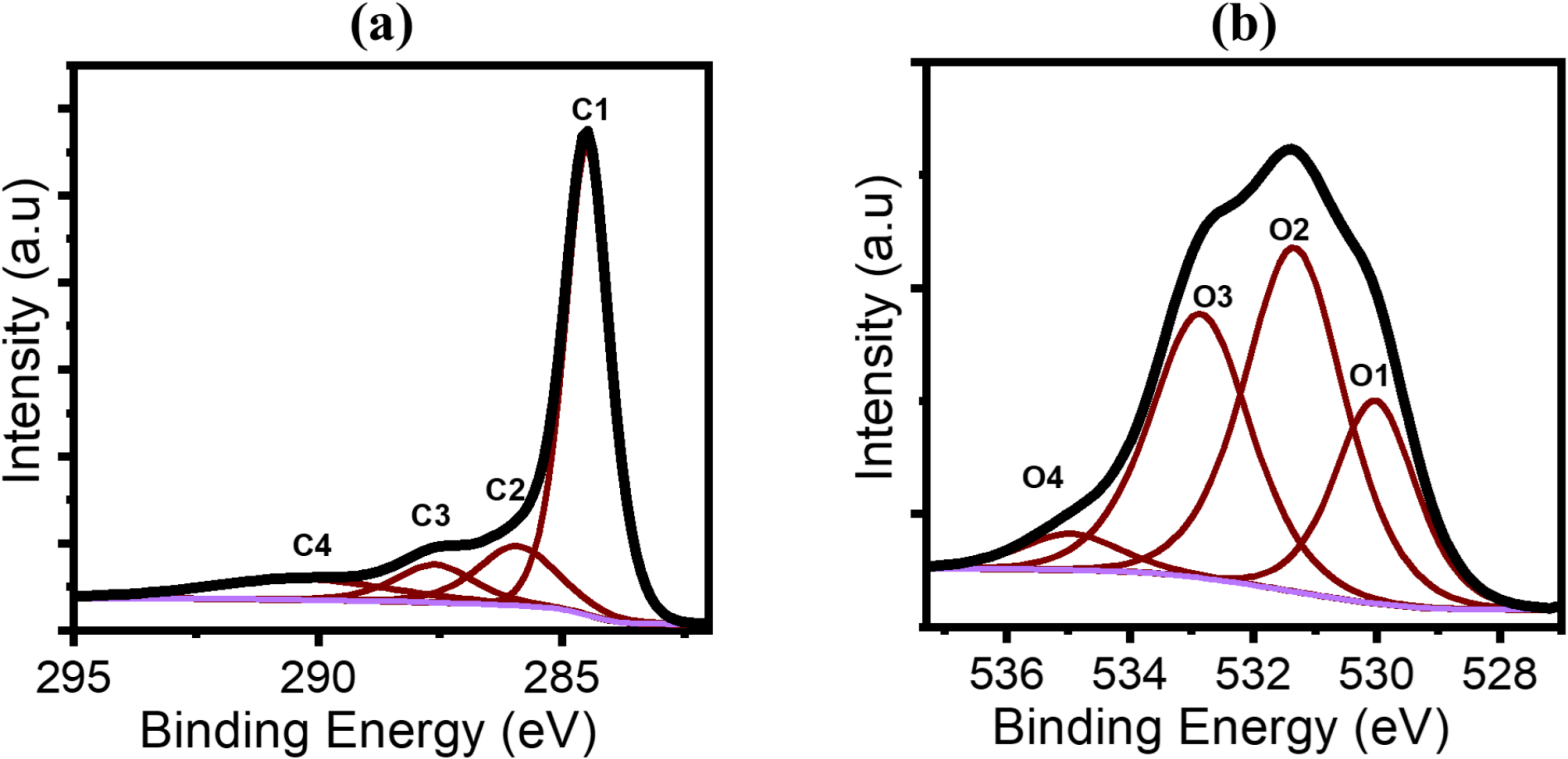
XPS spectra of (**a**) C1s and (**b**) O1s. The spectra are deconvoluted to four peaks.

The high-resolution XPS spectra of the C 1s region are shown in Fig. 5a. The deconvoluted C 1s peaks from GO are presented, with binding energies of 284.5, 285.92, 287.6, and 290.38 eV. These peaks correspond to the following carbon species: C1: C-C (60.8%), C2: C–O (13.64%), C3: C = O (8.65%), C4: O = C-O- (11.01%). The oxygen species were identified as follows (Fig. 5b): O1: O-C = O (1.2%), O2: C = O (2.5%), O3: C-OH (1.92%), and O4: C-O (0.26%) (see Table 6). The C/O ratio in graphene is typically very high due to the minimal presence of oxygen groups. However, in this oxidised compound, the C/O ratio was found to be 16, indicating an increase in the oxygen content after the preparation process, which introduces various oxygenated functional groups on the surface, enhancing the material’s surface reactivity^56–58^. However, the C/O ratio is not as low as ‘typical’ graphene oxide sheets (C/O about 2 to 4)^59,60^. This can be due to mild oxidation conditions and the presence of few-layer graphene, as it was observed under TEM in this study (Fig. 4b and c).

**Table 6:** XPS data: binding energies and atomic composition of GO.

Carbon	% C component	Binding energy (eV)	Oxygen	% O component	Binding energy (eV)	C/O ratio
C1	60.8	284.5	O1	1.2	530.02	16
C2	13.64	285.92	O2	2.5	531.33	
C3	8.65	287.6	O3	1.92	532.85	
C4	11.01	290.38	O4	0.26	534.96	

### Composite manufacturing

The GO was incorporated in biobased plastic (WG) and synthetic plastic (PA11) to ascertain the effect on their reaction-to-fire properties. PA11 and WG were chosen for this study due to their distinct properties and uses. PA11, a bio-based plastic, offers high strength, flexibility, and resistance to chemicals, making it suitable for demanding applications. WG, a natural polymer, provides a contrasting matrix to test, given its renewable nature and use in biodegradable materials. The combination of PA11 and wheat gluten allows for a comprehensive evaluation of GO’s effectiveness across different types of polymers, showing its potential to enhance fire safety in both synthetic and natural materials. All the samples were successfully formed and subjected to a cone calorimeter test.

#### Cone calorimeter test

**Fig. 6:**
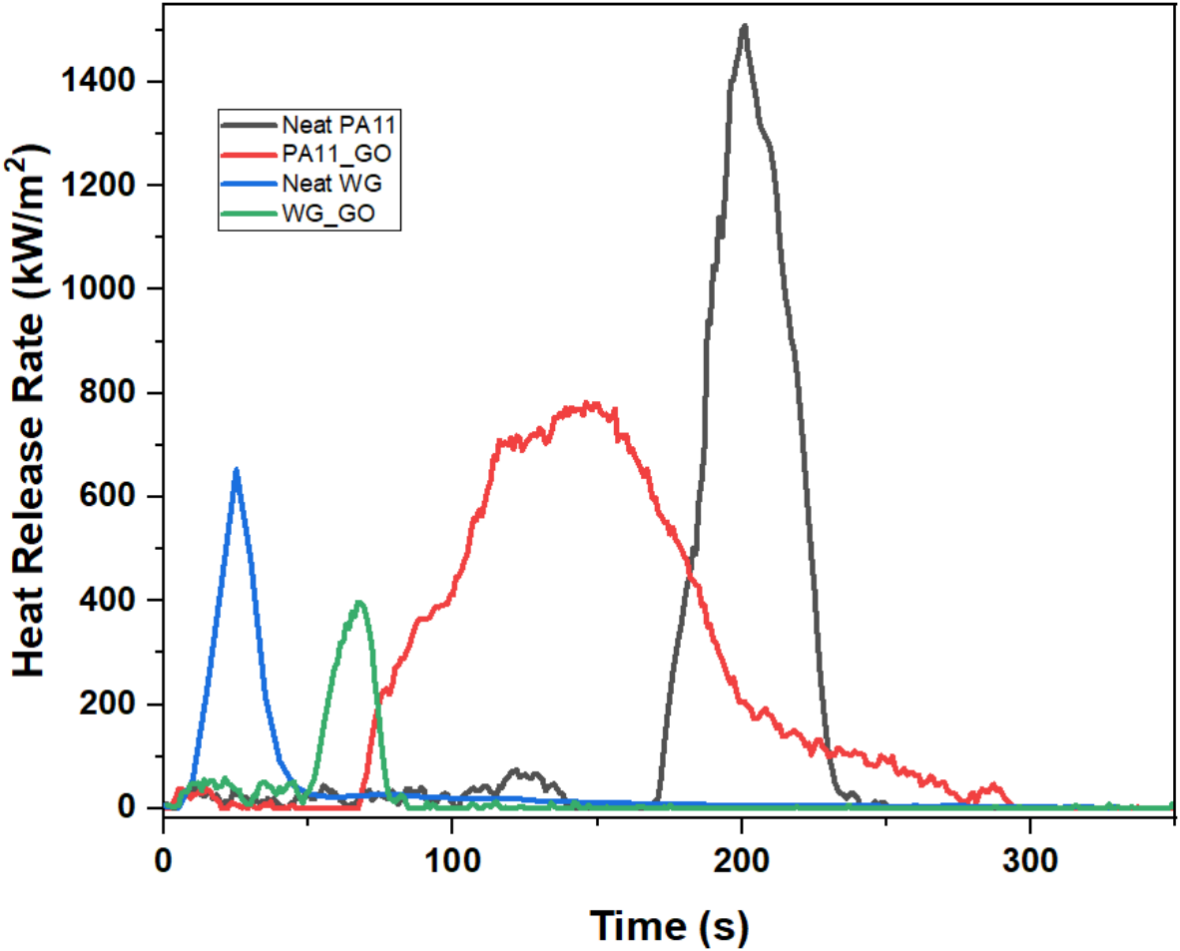
Heat release rate curves of samples tested in the cone calorimeter.

The heat release curves measured from the cone calorimeter are depicted in Fig. 6, while the reaction-to-fire properties of the samples are presented in Table 7. It is seen that the addition of 2wt.% GO significantly reduced the peak heat release rate (PHRR) of the samples, with a reduction of 42% in neat PA11 and 33% in neat WG. The two-dimensional structure of GO forms a surface barrier that prevents the release of volatile substances and flammable gases. Additionally, the high thermal conductivity of GO improves heat dissipation, limiting combustion propagation. It was also clearly seen during the experiment that the functional groups in GO serve as catalysts during thermal degradation, assisting in the creation of a char layer that protects and lowers flammability^61–63^.

The time to ignition (TTI) of neat PA11 decreased by 51% with the addition of GO, while that of WG increased by 168%. This could be attributed to the distinct thermal and combustion behaviours of the two materials, as well as the specific interactions between GO and each matrix. The dispersion and distribution of GO within the polymer matrix could also play a crucial role as it can impact how heat is conducted and how combustion products are generated, affecting the ignition behaviour differently. The increase in total heat release (THR) with the addition of GO shown in Table 7 reveals that the samples burnt for a long time due to the presence of a protective char layer restricting heat transfer to the sample.

The FPI (Fire Performance Index) and FIGRA (Fire Growth Rate) results provide insights into the fire behaviour of the studied materials. The FPI values, which express the time to ignition relative to the heat release rate, indicate that the addition of GO generally influenced the materials’ ignitability differently. The FPI values of neat PA11 and PA11_GO indicate no significant change in the fire performance, however, WG_GO exhibited a higher FPI, suggesting improved fire resistance with GO incorporation to WG. The FIGRA values, reflecting the rate of fire growth, highlighted the distinct effects of GO on combustion dynamics. Notably, neat PA11, PA11, and wheat gluten with GO exhibited lower FIGRA values, implying slower fire growth compared to neat WG.

The Flame Retardancy Index (FRI) is a dimensionless criterion developed to quantify the flame retardancy performance of polymer composites using cone calorimetry data^64^. This index integrates key fire reaction parameters: PHRR, THR, and TTI. The FRI is calculated by comparing the product of these parameters for a neat polymer to that of a composite containing flame retardant additives i.e.,

5 $$FRI=\left[ {\frac{{{{\left[ {\frac{{THR \times PHRR}}{{TTI}}} \right]}_{Neat}}}}{{{{\left[ {\frac{{THR \times PHRR}}{{TTI}}} \right]}_{Composite}}}}} \right]$$

An FRI value greater than 1 indicates an improvement in flame retardancy, while a value less than 1 suggests a reduction in performance. This approach allows for a standardised evaluation of various thermoplastic systems regardless of the type or loading level of the additive, providing a clear metric for assessing and comparing fire safety enhancements in different materials. The results in Table 7 suggest that GO has a more beneficial effect on the flame retardancy of wheat gluten compared to PA11. Future studies on GO composites will consider long-term stability factors, such as UV exposure and mechanical stress, to ensure consistent fire retardant properties over prolonged usage periods.

**Table 7:** Reaction-to-fire properties of samples obtained from cone calorimeter tests.

Sample	PHRR (kW/m^2^)	TTI (s)	TTPHRR (s)	THR (MJ/m²)	FPI (m² s/kW)	FIGRA (kW/m^2^ s)	FRI
Neat PA11	1508.3 ± 122	182 ± 0	199 ± 7	53.6 ± 6.2	0.12 ± 0.01	7.6 ± 0.5	–
PA11_GO	864.4 ± 114	88 ± 18	155 ± 11	76.7 ± 2	0.1 ± 0.02	5.6 ± 0.8	0.6 ± 0.2
Neat WG	652 ± 38	23 ± 4	25 ± 15	13.1 ± 7.0	0.04 ± 0.01	26.1 ± 15	–
WG_GO	433 ± 40	61.7 ± 15	67.7 ± 10	20.2 ± 10	0.14 ± 0.04	6.4 ± 1.1	2.6 ± 2

#### Flame retardant mechanism of GO

GO serves as an effective flame retardant in plastics through several physical mechanisms. Firstly, it establishes a barrier effect by forming protective layers in the polymer during combustion. Due to the high aspect ratio of GO platelets, these layers act as barrier to heat and mass transfer, significantly slowing the release of flammable gases and heat, thereby reducing the PHRR. Another reason that can be responsible for GO inducing fire-retardancy in plastics is the ‘tortuous path’ theory^65^ wherein the volatile gases resulting from polymer pyrolysis (as a result of high-temperature exposure) take time to evolve out through the barrier created by the stacked GO platelets. The slow evolution of flammable gases lowers the intensity of the feedback combustion and hence, increases fire-safety. Additionally, GO’s high thermal conductivity plays a vital role in dissipating heat away from the flame zone. This property can delay the time to reach peak heat release, though it may also cause faster initial heating, as observed in the reduced TTI for PA11 plastics. The dispersion of GO is also vital for its flame retardancy in bioplastics. Uniform dispersion ensures an even distribution throughout the polymer matrix, forming an effective barrier against flammable volatiles and oxygen diffusion during combustion. Conversely, poor dispersion leads to agglomeration, reducing GO’s effectiveness. These mechanisms collectively improve the flame retardancy of plastics containing GO. Some other previously unexplored mechanisms could be responsible for GO having a superior fire-retarding effect in plastics. This could be due to the relatively high electrical conductivity of highly carbonic materials, which affects the radiative heat transfer in a way that is beneficial for the fire performance^66^. Future investigations on fire safety of plastics using GO should delve into the aforementioned.

#### Integration of green chemistry principles and mechanisms involved in the facile Preparation of GO from waste biomass

**Fig. 7:**
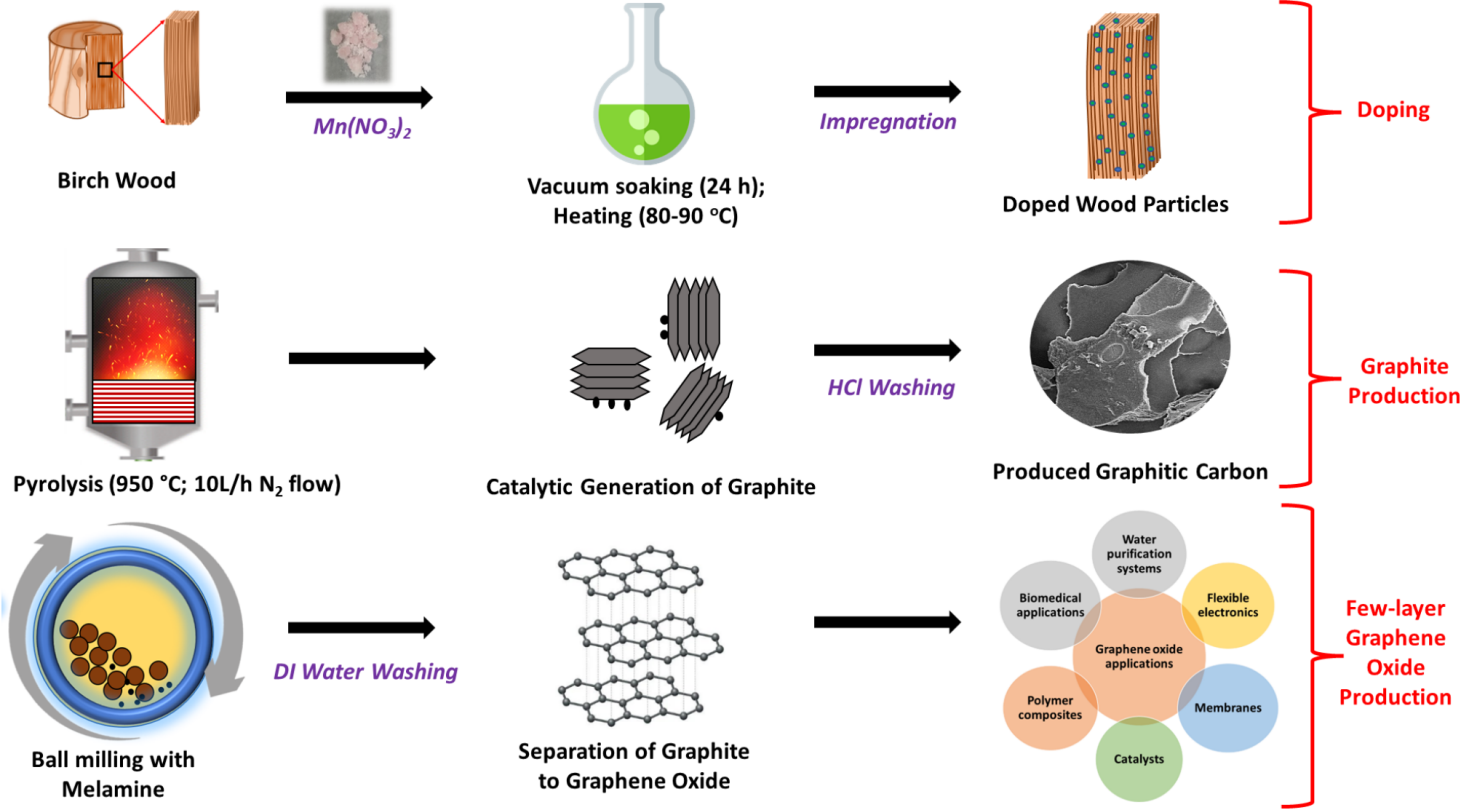
A schematic diagram showing the direct conversion of birch wood to GO.

Green chemistry has a set of 12 principles aimed at designing and developing chemical processes and products that minimise or eliminate the use and generation of hazardous substances. By integrating these principles, green chemistry strives to promote environmental sustainability, protect human health, and foster the development of innovative and environmentally friendly solutions for a more sustainable future. The current investigation satisfies six principles of green chemistry, namely, waste prevention, use of renewable resources, reduction of energy consumption, use of catalysts for efficiency, less hazardous synthesis, and reduction of derivatives. As shown in Fig. 7, in the present study, a renewable resource (i.e., waste prevention and valorisation), in the form of birch wood waste, was doped with manganese nitrate and pyrolysed at 950 °C to produce graphitic carbons without an intermediate amorphous carbon step, thus reducing the derivatives in the process. The doping of the wood particles with an effective catalyst (at low concentration) allowed the pyrolysis to take place at a lower temperature, 950 °C instead of temperatures greater than 1000 °C used in the conventional methods, which helped to promote energy efficiency. The entire synthesis was innocuous and no by-products, as derivates were formed.

Reports by several researchers show that metal nitrates are among the best catalysts to produce graphitic carbon^5,24^. At temperatures above 250 °C during the graphitisation process, the metallic nitrates decompose into the corresponding metal oxides. The pyrolytic decomposition of cellulose, lignin, etc. present in the wood to amorphous carbon occurs at temperatures between 300 and 600 °C. The metal oxides are then reduced at temperatures above 600 °C to get converted into the corresponding metal nanoparticles. The final step is the transformation of the amorphous carbon in contact with the metallic nanoparticles into more ordered carbon structures at temperatures above 700 °C. The extent of graphitisation is dependent on the amount of amorphous carbon that comes into contact with the metallic nanoparticles^1^. The carbon that is far from the metal nanoparticles remains amorphous proving the necessity for efficient penetration and homogenous distribution of the catalyst into the biomass. This increases the effectiveness of the graphitisation process resulting in the production of graphitic carbon. In order to increase the penetration of the metal catalyst (manganese nitrate) into the pores of the birch wood and consequently enhance the effectiveness of the catalytic graphitisation, the wood was soaked under vacuum and subsequently heated in the current study. The generated graphite was washed with dilute HCl to dissolve the salts produced as a result of catalyst decomposition. Lastly, the graphite was milled with melamine to separate the graphitic layers into graphene. Since the milling process was not inert, the graphene was oxidised as shown in the TEM and EDX results. The entire process, doping, graphitisation, and milling are shown in Fig. 7. The GO produced from this study can be used in various applications including polymer composite manufacturing, flexible electronics, environmental remediation, etc. Compared to conventional GO production, this waste biomass approach should have a reduced carbon footprint (for instance compared to the use of fossil-based precursors like coal) and lower energy demands due to the lower pyrolysis temperatures and renewable biomass source, making it a cost-effective and environmentally friendly alternative. While the method is highly scalable, potential industrial challenges include maintaining uniform doping across larger biomass quantities. Batch processing and automated control systems could address this for seamless industrial applications. Future improvements in GO synthesis could explore ultrasonic or mechanochemical methods, which are known for reducing energy consumption and have been successfully applied in low-energy catalytic processes (e.g., as discussed in the work of Osman et al.^67^).

## Conclusions

In this study, vacuum soaking, and heating at low temperatures (ca. 80–90 °C) were employed to efficiently dope a metal catalyst into the pores of wood followed by pyrolysis to develop graphitic carbon, which would be used subsequently to produce few-layer GO. This allows for the direct conversion of sustainable biomass (here birch wood waste) into graphitic carbon without the intermediary step of producing amorphous carbon. The effect of four different concentrations of the catalyst and processing times were investigated. Graphite was successfully produced from manganese nitrate doped wood pyrolysed at 950 ^o^C. Out of the four concentrations of catalysts (0.003, 0.005, 0.083, and 0.1 mol-metal/g-wood) and two residence times (1 and 2 h), the sample with 0.005 mol-metal/g-wood heated for 2 h had the highest graphitisation degree (70%) from X-ray diffraction studies and the least defects from Raman spectroscopy. Hence, this sample was chosen and milled in a planetary ball mill with melamine as a dispersant and surfactant. The few-layer GO was characterised using UV-Vis, EDX, XPS, and TEM. The fluorescence in UV-Vis showed a broad peak at 230 nm, similar to GO dispersions, EDX showed the presence of oxygen in the sample, and TEM revealed a semi-transparent structure with an irregular shape and variable layer thickness ranging from 3 to 8 analogous to that of GO. XPS analysis revealed the C/O ratio to be 16, indicating a carbon sample having structures akin to few-layer graphene with relatively low levels of oxygen functional groups. Based on the results, it can be concluded that few-layer GO has been successfully produced directly from waste wood without an intermediary amorphous carbon (biochar). This method’s low-temperature requirement and minimal use of external reagents offer significant cost advantages for scaling up, making it more feasible for industrial applications.

Furthermore, to explore the applications of the produced GO, 2wt.% was added to polyamide 11 and wheat gluten plastics and subjected to a cone calorimeter test. The results showed a 42% and 33% reduction in the peak heat release rate of the samples compared to the neat ones, which proved the effectiveness of the few-layer GO. The flame retardancy index of the plastics indicates that GO enhances the flame retardancy of wheat gluten more effectively than that of PA11. While effective, this approach has limitations in achieving uniform catalyst penetration across biomass and potential challenges in ensuring long-term stability of GO in diverse applications. Future research could address these by testing alternative catalysts, optimising doping parameters, and conducting extensive durability testing of GO-based composites under various environmental conditions. Additionally, alternative biomass feedstocks like agricultural residues could be explored as well as extended testing to assess GO’s durability in real-world applications.

## Data Availability

The XRD data of the current study is available in the HSU Cloud repository, https://cloud.hsu.ac.ir/s/cdZzjGqwXagToEW, other datasets generated during and/or analysed during the current study are available from the corresponding author upon reasonable request.
